# Tunable Spin dependent beam shift by simultaneously tailoring geometric and dynamical phases of light in inhomogeneous anisotropic medium

**DOI:** 10.1038/srep39582

**Published:** 2016-12-22

**Authors:** Mandira Pal, Chitram Banerjee, Shubham Chandel, Ankan Bag, Shovan K. Majumder, Nirmalya Ghosh

**Affiliations:** 1 Dept. of Physical Sciences, Indian Institute of Science Education and Research - Kolkata, Mohanpur 741 246, Nadia, West Bengal, India; 2 Raja Ramanna Centre for Advanced Technology, Indore 452013, India

## Abstract

Spin orbit interaction and the resulting Spin Hall effect of light are under recent intensive investigations because of their fundamental nature and potential applications. Here, we report an interesting manifestation of *spin Hall effect* of light and demonstrate its tunability in an inhomogeneous anisotropic medium exhibiting spatially varying retardance level. In our system, the beam shift occurs only for one circular polarization mode keeping the other orthogonal mode unaffected, which is shown to arise due to the combined spatial gradients of the geometric phase and the dynamical phase of light. The constituent two orthogonal circular polarization modes of an input linearly polarized light evolve in different trajectories, eventually manifesting as a large and tunable spin separation. The spin dependent beam shift and the demonstrated principle of simultaneously tailoring space-varying geometric and dynamical phase of light for achieving its tunability (of both magnitude and direction), may provide an attractive route towards development of spin-optical devices.

Spin orbit interaction (SOI) dealing with the coupling of spin and orbital degrees of freedom of massive (e.g., electron) and mass-less (e.g., photon) particles has led to several fundamental consequences in diverse fields of physics, ranging from atomic, condensed matter to optical systems. Since light can carry both spin (SAM, circular/elliptical polarization) and orbital angular momentum (OAM), on conceptual grounds, coupling and inter-conversion between the spin and orbital AM degrees of freedom of light is expected under certain circumstances. This leads to the SOI of light, and accordingly the evolution of polarized light in a trajectory mimics the SOI effect of a mass-less spin 1 particle (photon)^1^,^2^,^3^,^4^.

The SOI of light is typically manifested as two interdependent effects. The first one is the effect of the trajectory on the state of polarization of light, leading to the generation of spin (circular polarization)-dependent optical vortices, which usually takes place in cylindrically or spherically symmetric systems. The second one is the reverse effect of polarization on the trajectory of light, manifesting as a spin dependent shift of the trajectory of the light beam, known as the Spin Hall effect (SHE) of light^1^,^2^,^3^,^4^,^5^. This is usually associated with the breaking of the symmetry. The photonic SHE has been observed recently in various optical interactions^1^,^2^,^3^,^4^,^5^, each of which are discernable by important fundamental or applicative aspects. These are under recent intensive investigations because of their fundamental nature and also due to the fact that these are offering new opportunities for the development of spin-controlled photonic devices^1^,^2^,^6^,^7^. Note that generation of geometric phases (and its gradient) and subsequent conservation of the total angular momentum of light is intimately associated with the optical SOI phenomena. There are two variants of the geometric phase that contribute to the SOI phenomena–the spin redirection Berry phase and the Pancharatnam-Berry (PB) geometric phase. SOI effects produced via spin redirection Berry phase (e.g., in tight focusing of fundamental or higher order Gaussian beams, scattering from micro/nano systems, reflection/refraction at dielectric interfaces etc.), is generally weak^1^,^2^,^3^,^4^. Despite the fact that such tiny SHE (typically in the sub-wavelength domain) has already been explored for selected applications in nanophotonics^5^,^8^, development of methods and systems for enhancing this effect is highly desirable for numerous potential applications. Recent efforts towards enhancing the SOI and the SHE effects therefore exploited the PB geometric phase in inhomogeneous anisotropic medium, which can be considerably stronger^6^,^7^,^9^,^10^. However, realization of tunable spin-dependent splitting of light beam remains to be an outstanding challenge. Here, we report a *spin dependent* beam shift and demonstrate its full tunability in an inhomogeneous anisotropic medium exhibiting user-controlled spatially varying retardance level. Unlike other variants of SHE, here the beam shift occurs only for one circular polarization mode keeping the other orthogonal mode unaffected, which is shown to arise due to the combined spatial gradients of the geometric and dynamical phases of light. In a simple yet elegant system of a twisted nematic liquid crystal-based spatial light modulator (SLM), we demonstrate that one can simultaneously generate desirable spatial gradients of both the geometric and the dynamical phases of light to produce such spin dependent beam shift in a regulated fashion. The effect is eventually manifested as a spin-dependent splitting of input linearly polarized beam, where the constituent two orthogonal circular polarization modes evolve in different trajectories leading to a large and tunable spin separation. The demonstrated principle of simultaneously tailoring space-varying geometric and dynamical phase of light for achieving the spin dependent beam shift and its tunability (of both magnitude and direction), may provide an attractive route towards development of spin-optical devices for spin-controlled photonic applications^7^.

## Theory

### Spin Specific Beam shift

Temporal evolution of geometric phase is known to manifest as an input polarization-dependent shift of the frequency (ω) of light^11^,^12^,^13^. It has been shown previously that the spatial analogue of this effect in a transversely inhomogeneous anisotropic medium is related to the SOI of light^14^,^15^,^16^. Here, we describe a simple yet intriguing effect associated with the geometric phase gradient in an inhomogeneous anisotropic medium. A Gaussian beam propagating along the z-direction of an inhomogeneous (along the transverse x/y direction) anisotropic medium is associated with transverse momentum components *(k*_*⊥*_ = *k*_*x*_ and *k*_*y*_). A space varying Pancharatnam-Berry (PB) geometric phase 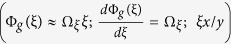 manifests as input circular polarization (SAM) dependent shift in the transverse momentum distribution (Δ*k*_*⊥*_) of the beam. If one further introduces a spatial gradient of dynamical phase 
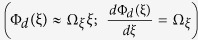
, it may happen that for one circular polarization mode, the two spatial gradients cancel out to yield no net shift. For the other orthogonal mode, on the other hand, they may yield an accumulated shift of the beam centroid.

For a paraxial Gaussian beam, we neglect small longitudinal field component and represent the transverse components as


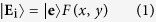


where 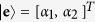 is the Jones vector of the homogeneously polarized input beam and *F*(*x, y*) is its Gaussian envelop. On propagation through the inhomogeneous anisotropic medium, it acquires space varying dynamical (Φ_*d*_(*ξ*)) and PB geometric phase (Φ_*g*_(*ξ*)). The output field can be written as


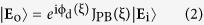


here, J_PB_(*ξ*) is a 2 × 2 matrix representing the effect of the PB geometric phase Φ_*g*_(*ξ*)^16^.

For input RCP 
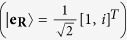
 and LCP 
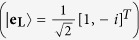
 states, the output field becomes


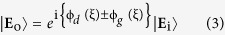


here, ± correspond to input RCP and LCP states, respectively.

The expectation values of the transverse co-ordinates and the momenta of the output beam can then be calculated as


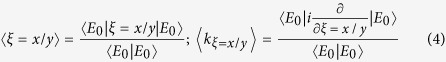


For input RCP and LCP states, the quantities *ξ* = *x*/*y* and *k*_*ξ *= *x*/*y*_ can be determined by using eq. (3) in eq. (4). While, *ξ* = *x*/*y*vanishes, yielding no net co-ordinate shift, the momentum shift becomes non-zero and determined by Φ_*d*_(*ξ*) and Φ_*g*_(*ξ*). When the two phases have equal gradient 
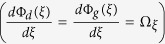
, the momentum domain shifts for input RCP and LCP states become


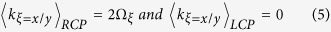


We shall subsequently demonstrate that equal spatial gradients of geometric and dynamical phases can indeed be produced in a twisted nematic liquid crystal-based SLM by modulating its pixels with user controlled grey level distributions (*n*). We now turn to the modeling of the evolution of PB geometric phase and dynamical phase of light in such system.

### Pancharatnam-Berry (PB) geometric phase and dynamical phase in twisted nematic liquid crystal layers

When polarized light propagates through an anisotropic (birefringent) medium, it acquires both PB geometric phase and dynamical phase. The dynamical phase for a linear birefringent medium is determined by the extraordinary and ordinary refractive indices (*n*_*e*_ and *n*_*o*_) and consequently it also depends upon the magnitude of linear retardance *δ* (defined as 
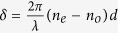
, where *d* is the path length and λ is the wavelength). The PB geometric phase in such birefringent medium, on the other hand, is determined by the orientation angle of the anisotropy axis^15^. Thus, in principle, one can produce equal spatial gradients of the dynamical phase and PB geometric phase in an inhomogeneous birefringent medium by controllably (and simultaneously) changing the magnitude of linear retardance *δ* and the orientation angle of the anisotropy axis in the transverse plane (*x/y*-plane, with z being the propagation direction of light). In case of a twisted nematic liquid crystal-based spatial light modulator (SLM), the system is slightly more complex. The SLM comprises of many layers of liquid crystals exhibiting linear birefringence with the axis of anisotropy of each layers twisted by an angle. The net anisotropy effects of such twisted birefringent layers manifest as both linear retardance and optical rotation. This optical rotation effect has geometric origin. In such system, while the total dynamical phase is primarily determined by the total accumulated linear retardance, the PB geometric phase is determined by the effective optical rotation. Since, the magnitudes of the linear retardance and optical rotation can be controlled by changing the grey level values (*n*) in the SLM, one can controllably generate both geometric and dynamical phases of light, as described below.

The evolution of both the phase and the polarization of light in twisted nematic liquid crystal layers can be modeled using the following equation^17^


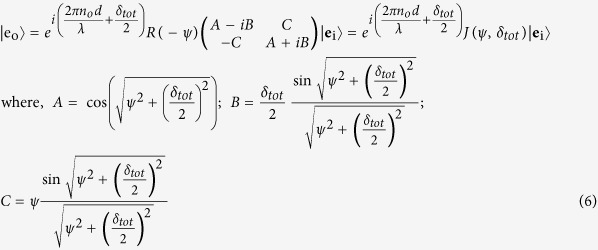


here, 
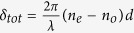
 is the total linear retardance, *n*_*e*_ and *n*_*o*_ are the extraordinary and ordinary refractive indices, *ψ* is the twist angle, *J* is the Jones matrix of the system containing the 2 × 2 rotation matrix *R*(−*ψ*). The evolution of polarization in SLM can also be alternatively modeled using the effective Jones matrix (*J*_*eff*_) as a sequential product of matrices of an equivalent linear retarder (*J*_*reta*_, with effective linear retardance *δ*_*eff*_and its orientation angle *θ*_*eff*_) and an effective optical rotator (with optical rotation *ψ*_*eff*_)^18^


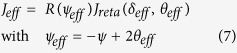


The dynamical phase (for input LCP/RCP states) is clearly given by the phase factor in eq. (6)


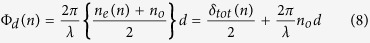


here, *n* is the value of grey level(s) applied to a twisted nematic liquid crystal-based SLM (which is used to observe the spin specific beam shift in our experiments, as described subsequently). Henceforth, we only consider the first term in eq. (8), since, the grey level dependent part is only relevant to the effect. The matrix *J* (in eq. 6) is free from dynamical phase 
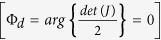
19 and the corresponding PB geometric phase encoded in it, can be determined using the Pancharatnam connection^19^,^20^,^21^, for input RCP (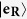) and LCP (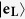) states as


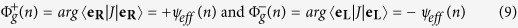


As apparent from the above theoretical treatment, when polarized light propagates through twisted nematic liquid crystal layers, it acquires both dynamical and PB geometrical phases. The PB geometric phase arises here due to the non-cyclic polarization evolution^19^,^21^ in the twisted birefringent structure (due to the twist along the longitudinal direction). As shown in Eq. 9, the resulting PB geometric phase is determined by the effective optical rotation *ψ*_*eff*_ parameter, which has geometrical origin. The total dynamical phase, on the other hand, is determined by the total linear retardance *δ*_*tot*_ parameter (see Eq. 8). Importantly, since both the *ψ*_*eff*_ and the *δ*_*tot*_ parameters can be controlled by changing the grey level values (*n*) in the SLM, both the dynamical and the PB geometric phases can be simultaneously generated in a controlled manner. One can thus produce spatial gradients of the dynamical and the PB geometric phases by creating grey level gradient in the SLM along one chosen linear direction (*x or y*) (see Methods). However, for this purpose, one needs to first experimentally determine the dependence of the *ψ*_*eff*_(*n*) and *δ*_*tot*_(*n*) parameters on the grey level value *n*. For producing equal spatial gradients of the two phases, one then needs to choose the range of grey levels for which the gradients 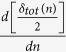 and 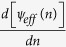 are nearly equal.

Note that eqs (7, 8 and 9) in combination with experimental Mueller matrix^22^,^23^ measurements, can be used to determine both the geometric and the dynamical phases of light. For this purpose, full 4 × 4 Mueller matrices *M* can be recorded from the SLM having uniform grey level (*n*) addressing. The effective linear retardance *δ*_*eff*_(*n*) and optical rotation *ψ*_*eff*_(*n*) parameters can be determined from the elements of *M*, by representing it as a product of basis matrices of an equivalent linear retarder and rotator (Jones → Mueller matrix conversion of eq. 7) (see Supplementary Information)^23^. The twist angle of the SLM (*ψ*) can also be determined separately^18^. Using these set of parameters, one can determine the magnitudes of total retardance *δ*_*tot*_(*n*) from the relationship connecting them (derived from the equivalence of eqs 6 and 7)^18^.


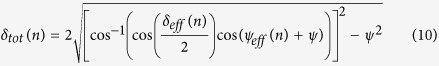


Thus obtained *δ*_*tot*_(*n*) and *ψ*_*eff*_(*n*) parameters may finally be used to determine the values of Φ_*d*_(*n*), 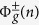, and the resulting total phase [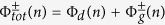, ± corresponding to input RCP and LCP states, respectively].

In what follows, (*i*) we experimentally demonstrate the spin dependent beam shift and its tunability in an inhomogeneous anisotropic medium; (*ii*) We then determine both the space varying dynamical and the geometric phases in such system to demonstrate the underlying principle; (*iii*) Finally, we show large spin dependent splitting of input linearly polarized beam.

## Results and Discussion

### Tunable Spin Specific Beam Shift in inhomogeneous anisotropic medium

**Figure 1:**
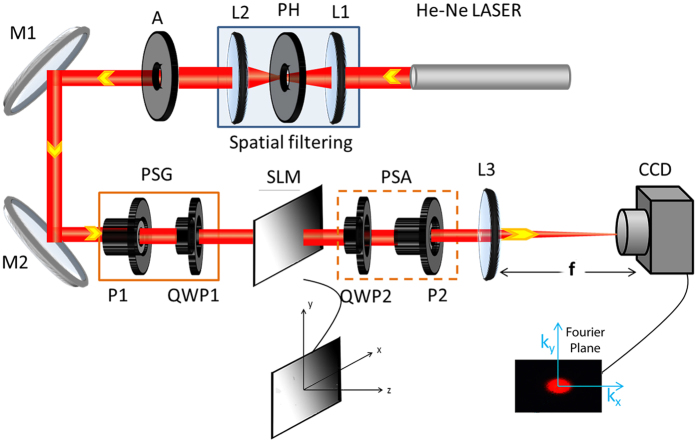
Schematic of the experimental arrangement for observing tunable spin specific beam shift and spin-dependent splitting of light beam. The polarization state generator (PSG) and the polarization state analyzer (PSA) units comprising of a fixed linear polarizer and a rotatable quarter waveplate, are used to generate and analyze desirable polarization states of light. L_1_, L_2_, L_3_: Lenses; P_1_, P_2_: linear polarizers; QWP_1_, QWP_2_: quarter waveplates, SLM: spatial light modulator, M_1_, M_2_: mirrors, PH: pinhole, A: aperture here. While studying the spin specific beam shift, the PSG unit was used to sequentially generate RCP and LCP polarization states and the PSA unit was removed. For observing the spin-dependent splitting of light, the PSG was used to generate linear polarization state, and the transmitted beam was sequentially analyzed via the RCP and LCP analyzer states of the PSA. The same system was also employed to record Mueller matrices from the SLM.

**Figure 2:**
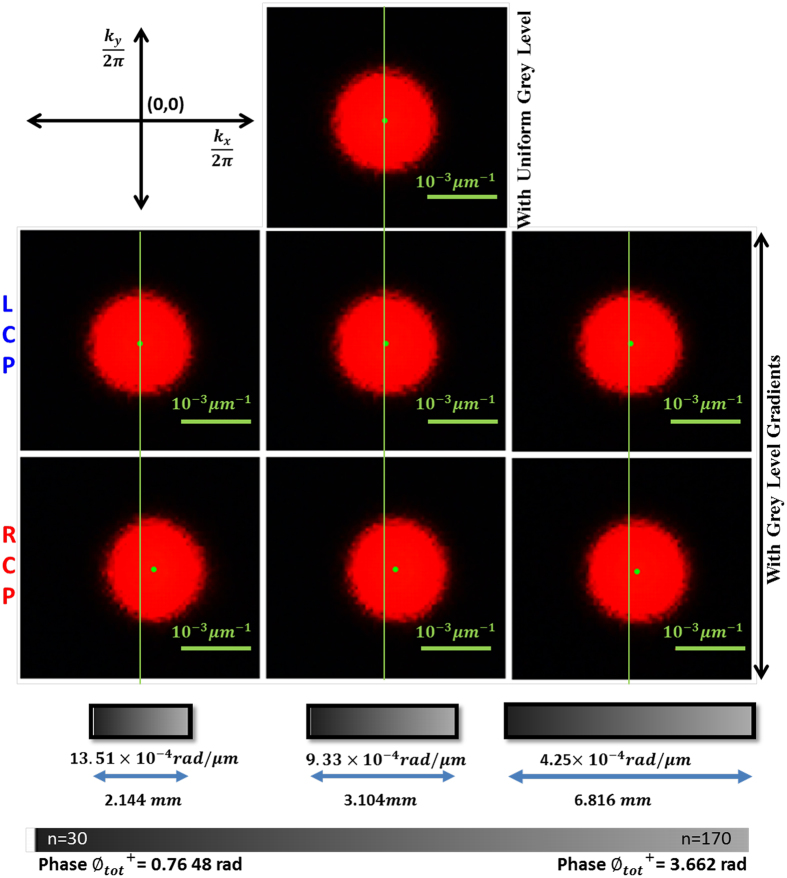
Experimental observation of tunable spin specific beam shift. Transverse momentum distribution (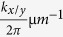) of the transmitted beam for input LCP (middle panel) and RCP (bottom panel) states, for three representative spatial gradients of grey levels 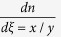 = 0.0205, 0.0451, 0.0653 bit/μm (right to left). Grey levels (values *n* = 30–170) are shown using color bar and the spatial dimensions over which these were applied, are noted. Result for a uniform grey level distribution is displayed as reference (top panel). While, the beam centroid for input RCP state exhibits large and systematic shift with increasing 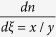, that for LCP state does not exhibit any appreciable shift, demonstrating spin specific beam shift and its tunability. The grey level dependence of total phase experienced by RCP state 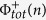 (shown using color bar) and the noted phase gradients are based on determination of geometric and dynamical phases, results of which are presented subsequently.

The inhomogeneous anisotropic medium was realized by modulating the pixels of a twisted nematic liquid crystal-based SLM by user controlled grey level (*n*) distributions (see Methods). Making use of the experimental arrangement (Fig. 1) and using varying spatial gradient of the grey levels in the SLM, spin dependent beam shift (shift in transverse momentum distribution 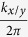) and its tunability is demonstrated in Fig. 2. The momentum domain beam shift for the input RCP state increases systematically with increasing spatial gradient of grey level (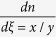). Remarkably, the shift of the beam centroid (momentum shift manifested as a shift of the centroid) becomes as large as ~ 25.16 μm for the highest applied spatial gradient 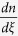 = 0.0653 bit/μm (varied between 11.56–25.16 μm for 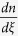 = 0.0205–0.0653 bit/μm). The beam centroid for input LCP state, on the other hand, does not exhibit any appreciable shift (Fig. 2). While, these results are for applied grey level gradient along the x-direction, similar results were also obtained for that applied along the y-direction. We now proceed to determine the space varying PB geometric phase and dynamical phase and relate these to the observed effect (the values of the spatial gradients of total phase 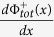 for the RCP state noted in Fig. 2, are based on these, as described below).

### Determination of PB geometric phase and dynamical phase

**Figure 3:**
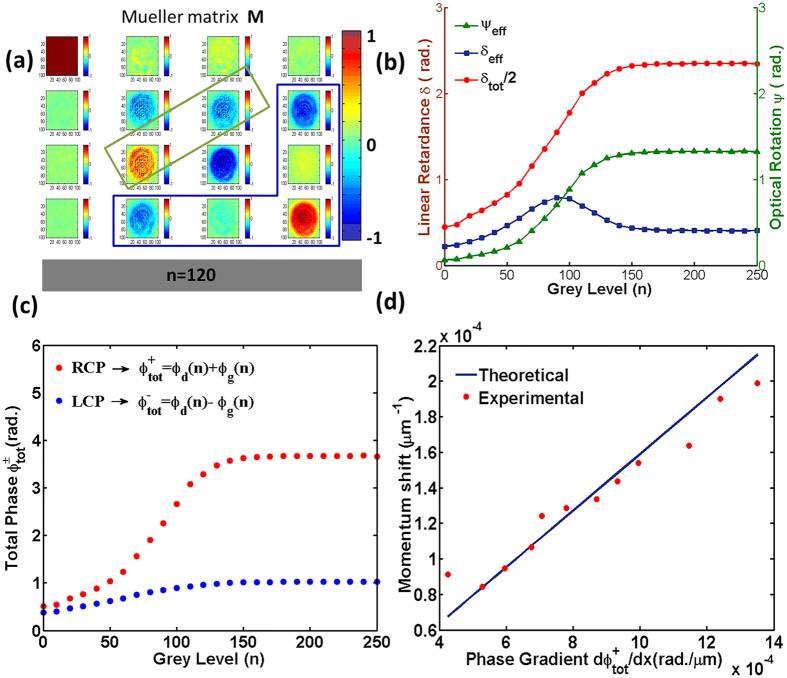
Experimental determination of PB geometric phase and dynamical phase of light. (**a**) Illustrative example of Mueller matrix recorded from the SLM having a uniform grey level addressing of *n* = 120. (**b**) The variation of the polarization parameters, effective linear retardance *δ*_*eff*_(*n*) (blue square), optical rotation *ψ*_*eff*_(*n*) (gree. nriangle) and total retardance *δ*_*tot*_(*n*) (red circle) (line is guide for eye). (**c**) The corresponding variation of the total (dynamical + geometric) phase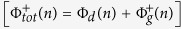 for input RCP (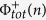, red circle) and LCP (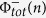, blue circle) states. (**d**) Comparison of the experimental shifts for RCP state (red circle) and corresponding theoretical predictions (blue line). Gradual increase of 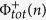 (for *n* = 30–170 used in the experiments of Fig. 2) for RCP statresponding nligible variation for LCP state and the agreement between the experiments and theory provide concrete evidence of the underlying principle of the spin specific beam shift.

The geometric and the dynamical phases of light were determined from experimental Mueller matrix measurements in combination with the treatment outlined previously. Mueller matrices were recorded from the SLM having different uniform grey level (*n*) addressing (see Methods and Supplementary Information). The twist angle of the SLM was separately determined to be *ψ* = *π/2*^18^. The results of the Mueller matrix measurements, determination of the medium polarization parameters [*δ*_*eff*_(*n*), *ψ*_*eff*_(*n*) and *δ*_*tot*_(*n*)], and the corresponding dynamical (Φ_*d*_(*n*)) and geometrical phases (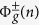) of light are summarized in Fig. 3. A typical Mueller matrix M recorded from the SLM for *n* = 120 is shown in Fig. 3a, the elements are represented in normalized unit (normalized by the M_11_ element). The normalized values of the elements (between −1 to +1) are shown using the color bar. The Mueller matrices show characteristic features of pure retarders (Fig. 3a, and Supplementary Information). Specifically, the signatures of the effective linear retardance (*δ*_*eff*_(*n*)) is manifested in considerable magnitudes of the M_24_/M_42_ and M_34_/M_43_ elements. The corresponding signature of effective optical rotation (*ψ*_*eff*_(*n*)) is reflected as a difference in the magnitudes of the M_23_ and M_32_ elements. The weak magnitudes and negligible variation of the elements of the 1^st^ row and the 1^st^ column of the matrices (M_12_/M_21_, M_13_/M_31_, M_14_/M_41_ elements, which encode polarization diattenuation effect^22^,^23^) with varying *n* implies negligible polarization dependent intensity modulation effect. The δ_eff_(*n*) and the ψ_eff_(*n*) parameters were subsequently determined from the recorded Mueller matrices (see Supplementary Information). The total linear retardance parameter *δ*_*tot*_(*n*)was then determined using the vales for δ_eff_(*n*)and the ψ_eff_(*n*) in Eq. (10). From the estimated medium polarization parameters (*δ*_*eff*_(*n*), *ψ*_*eff*_(*n*) and *δ*_*tot*_(*n) in* Fig. 3b) it appears that for the range of grey levels (*n*=30–170) the *ψ*_*eff*_ and the *δ*_*tot*_ parameters have nearly same gradient (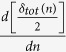 ~ 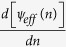). The resulting total phase (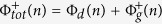, derived using eqs 8, 9 and 10 and Fig. 3c) underscore the key feature pertinent to the observed spin dependent beam shift (Fig. 2). While for input RCP state, 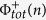 increases gradually with increasing *n* (for the range *n* ≈ 30–170 used in the experiments and accordingly displayed in Fig. 2), the corresponding variation for input LCP state (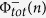) is rather weak and negligible. The small (but non-zero) total phase gradient for input LCP state implies that 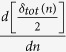 is nearly but not exactly equal to 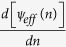 for the SLM. It should be noted that the determined polarization parameters (*δ*_*eff*_(*n*), *ψ*_*eff*_(*n*) and *δ*_*tot*_(*n*)) and the corresponding estimates for the dynamical and the PB geometric phases are subject to small uncertainties (deviation from ideal values) due to the various approximations used in the theoretical model. Never-the-less, this extremely small spatialradient of total phase for input LCP state (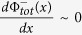) did not lead to measurable shift in the beam centroid for input LCP state (Fig. 2), which provides experimental support towards the validity of the theoretical model. Comparison of the experimental momentum domain beam shifts for the input RCP state and the corresponding theoretical predictions (using the results of Fig. 3c in eq. 5) shows reasonable agreement (Fig. 3d). For the theoretical predictions, an approximated linear dependence of 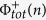 with *n* (for *n* ≈ 30–170 in Fig. 3c) was assumed and the spatial dimensions (over which the grey levels were applied) were duly considered (the values of the spatial gradients 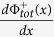 noted in Fig. 2 are based on this approximation). Incorporation of the exact dependence of 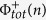 did not lead to significant differences in the predicted trends and absolute values. These results provide concrete evidence of the underlying principle– while for input RCP state, the accumulation of the spatial gradients of the dynamical and the geometrical phases lead to a large shift of the beam-centroid, the spatial gradients nearly cancel out to yield no appreciable shift for the input LCP state.

### Spin dependent splitting of input linearly polarized light beam

**Figure 4:**
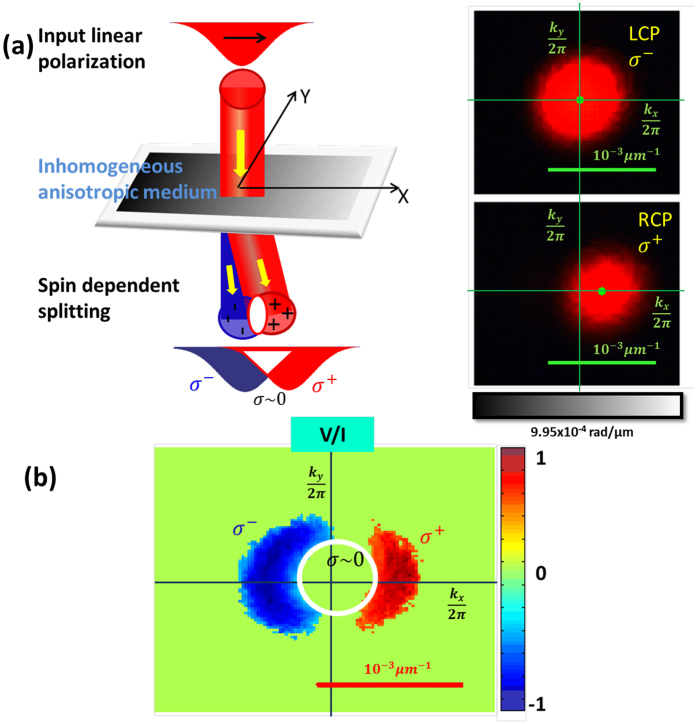
(**a**) Spin dependent splitting of input linearly polarized light in an inhomogeneous anisotropic medium exhibiting nearly equal spatial gradient of geometric and dynamical phases. One of the constituent circular polarization mode (RCP, noted as σ^+^) experiences a large and tunable momentum domain shift (manifested as a shift of the beam centroid in the detection plane, shown in bottom panel), the other orthogonal mode (LCP, σ^−^, top panel) evolves in the same trajectory. The results are displayed for a spatial gradient of the total phase of 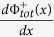 = 9.95 × 10^−4^ rad/μm (as experienced by the RCP mode). (**b**) The spin separation is shown by the spatial distribution of the circular polarization descriptor Stokes Vector element *V/I (V* is the difference in intensities between the RCP and LCP components and *I* is the sum of the two, the total intensity).

The spin dependent beam shift is eveually manifested as a spin dependent splitting of input linearly polarized beam. Like in photonic SHE, the constituent two orthogonal circular polarization modes evolve in different trajectories, leading to a large (and tunable) spin separation (shown in Fig. 4a,b). Figure 4a displays the transverse momentum distribution (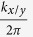
μm^−1^) of the transmitted beam (for input linearly polarized beam) after passing through the left (LCP) and right (RCP) circular analyzers. As can be seen, while the constituent RCP mode (noted as σ^+^) experiences a large and tunable momentum domain shift (manifested as a shift of the beam centroid in the detection plane, shown in the bottom panel of Fig. 4a), the other orthogonal mode (LCP, σ^−^, top panel) evolves in the same trajectory. The spin dependent splitting of input linearly polarized light is evident in the spatial distribution of the circular polarization descriptor Stokes Vector element 
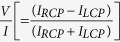
 (Fig. 4b), which shows spatially separated regions of opposite circular polarization states. We note that this spin dependent beam shift is perfectly discernible from other variants of SHE^1^,^2^,^3^,^4^,^5^,^6^,^8^,^9^,^14^,^24^ in that–(*a*) only one circular polarization mode experiences the shift, the other orthogonal mode evolves in the same trajectory, (*b*) the magnitude as well as the direction of the splitting is completely tuneable. Moreover, unlike other variant of the momentum domain beam shifts (angular Goos-Hänchen and the angular Imbert-Federov shifts^16^,^25^), the shift is independent of the beam waist parameter and is exclusively determined by the dynamical and the geometric phase gradients.

To summarize, we have observed an interesting manifestation of spin dependent momentum domain beam shift of light and demonstrated its tunability in an inhomogeneous anisotropic medium. The effect is manifested as a shift of the beam centroid for one circular polarization mode whereas the other orthogonal mode remains unaffected and evolves in the same trajectory. This is shown to arise due to the combined spatial gradients of the PB geometric phase and dynamical phase of light. It is pertinent to emphasize here that the SOI and the SHE effects in inhomogeneous anisotropic medium reported so far, deal with systems having spatially varying axis of retardance (and with constant magnitude of retardance), which leads to generation of space varying geometric phase alone^9^,^15^,^24^,^26^. In contrast, as demonstrated here, if one introduces a spatially varying magnitude of retardance, one may simultaneously generate space varying PB geometric phase and dynamical phase of light in a regulated manner to produce momentum domain beam shift for one circular polarization mode only. While the effect is demonstrated in a twisted nematic liquid crystal-based system, albeit with a relatively smaller spatial gradient (limited by the spatial dimension of the pixels), the principle can be extended to a wide class of anisotropic nano-optical systems^6^,^7^,^26^,^27^,^28^, wherein the phase gradients can be enhanced by several orders of magnitude to produce giant spin dependent beam shift. For example, space varying anisotropy effects can be tailored in specially designed plasmonic nano structures^24^,^26^,^28^,^29^,^30^ to produce such effects. We are currently expanding our investigations in this direction. In general, the remarkable simplicity of the approach of simultaneously tailoring spatial gradient of geometric and dynamical phases of light to produce such dramatic spin dependent beam shift may provide an attractive route towards development of spin-controlled photonic devices for the generation, manipulation and detection of spin-polarized photons.

## Methods

A schematic of the experimental system is shown in Fig. 1. Fundamental Gaussian (TEM_00_) mode of 632.8 nm line of a He–Ne laser (HRR120-1, Thorlabs, USA), is spatially filtered, collimated (using Lens, Pinhole and aperture assembly), and made incident on a transmissive spatial light modulator (SLM). The transverse momentum distribution (*k*_*⊥*_ = *k*_*x*_ *and k*_*y*_) of the transmitted beam is imaged into a CCD camera (Micro Publisher 3.3, Qimaging, 2048 × 1536 square pixels, pixel dimension 3.45 μm). The polarization state generator (PSG) and the polarization state analyzer (PSA) units are used to generate and analyze desirable polarization states of light. The PSG unit comprises of a fixed Glan-Thompson linear polarizer (P_1_, GTH10M, Thorlabs, USA) and a rotatable quarter waveplate (QWP_1_, WPQ10M-633, Thorlabs, USA) mounted on a computer-controlled rotational mount (PRM1/MZ8,Thorlabs, USA). The PSA unit essentially consists of a similar arrangement of fixed linear polarizer (P_2_) and a rotatable quarter waveplate (QWP_2_), but positioned in a reverse order.

In order to observe tunable spin specific beam shift and spin-dependent splitting of light beam, the inhomogeneous anisotropic medium (having spatially varying birefringence level) was realized by modulating the pixels of the SLM by user controlled grey level (*n*) distributions. Desirable grey level gradient was created along one chosen linear direction (x or y) using a range of grey level values between *n* = 30 to 170. For this range the grey level gradient of the total retardance parameter was nearly equal to the corresponding gradient of the effective optical rotation parameter (
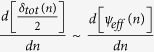
, Fig. 3b). This choice of the range of *n* enabled us to produce equal spatial gradients of the dynamical and the PB geometric phases in the SLM by creating grey level gradient in the SLM along one chosen linear direction (*x or y*). Variable spatial gradient of grey levels was achieved by accommodating this within variable spatial dimensions (Δ*x* = 2.144 mm–6.816 mm, limited by the beam spot size and the transverse width of the SLM) of the relayed image to the SLM. Since, the spatial dimension Δ*x* was varied in equal steps keeping the total range of phase (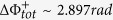, corresponding to *n* = 30 to 170, Fig. 3c) fixed, the spatial gradient of the total phase 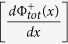 was not changed in equal steps (as apparent from Fig. 3d). While studying the spin specific beam shift, the PSA unit was removed and the PSG unit was used to sequentially generate RCP and LCP polarization states. For observing the spin-dependent splitting of light, the PSG was used to generate linear polarization state, and the transmitted beam was sequentially analyzed for RCP and LCP analyzer basis states of the PSA. In either case, the SLM, acting as an inhomogeneous anisotropic medium, was positioned at the front focal plane of the Fourier transforming lens L3 (focal length f), and the CCD camera was placed at its back focal (Foier) plane. In this configuration, the recorded intensity distribution at the CCD (*x*′, *y*′) plane (Fourier plane) represented the transverse momentum (spatial frequency) distribution 
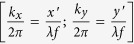
. The centroid of the transverse momentum distribution *k*_*x*/*y*_ was subsequently determined. Measurements were first performed by giving uniform grey level distribution in the SLM, for which there was no appreciable shift in the beam centroid between LCP/RCP input beams. The momentum domain spin specific beam shifts (from the SLM having desirable spatial gradient of grey level) were then quantified by taking the aforementioned measurement as a reference.

The same system was also used to record the Mueller matrices of the SLM having different uniform grey level addressing. The Mueller matrix measurement strategy is based on sequential generation (using the PSG unit) and analysis (by the PSA unit) of four optimized elliptical polarization states^31^,^32^. A series of sixteen intensity measurements (images) were performed by sequentially changing the orientation of the fast axis of the quarter waveplates of the PSG unit and that of the PSA unit, to four optimized angles 35°, 70°, 105° and 140° (representing four optimized generator elliptical basis states and corresponding four analyzer basis states) (see Supplementary Information). During these measurements, the axis of the polarizer (P_1_) in the PSG was fixed along the laboratory horizontal direction and that (P_2_) in the PSA was kept along the laboratory vertical direction. These sixteen intensity measurements were then combined to generate the system Mueller matrix following the approach described in ref. 30. Eigenvalue calibration of the system ensured high accuracy of Mueller matrix measurement (accuracy ~0.01 in normalized matrix elements)^30^.

## Additional Information

**How to cite this article**: Pal, M. *et al*. Tunable Spin dependent beam shift by simultaneously tailoring geometric and dynamical phases of light in inhomogeneous anisotropic medium. *Sci. Rep.* **6**, 39582; doi: 10.1038/srep39582 (2016).

**Publisher's note:** Springer Nature remains neutral with regard to jurisdictional claims in published maps and institutional affiliations.

## Supplementary Material

Supplementary Information
