# Supplementary material for: Tunable Spin dependent beam shift by simultaneously tailoring geometric and dynamical phases of light in inhomogeneous anisotropic medium
Source: Sci Rep. 2016 Dec 22;6:39582. doi: 10.1038/srep39582 (PMC5177887; doi:10.1038/srep39582)
Supplement: Supplementary Information [file srep39582-s1.pdf]

Supplementary Information

Tunable Spin dependent beam shift by simultaneously tailoring geometric and dynamical phases of light in inhomogeneous anisotropic medium

Mandira Pal[1], Chitram Banerjee[1], Shubham Chandel[1], Ankan Bag[1],
Shovan K Majumder [2] and Nirmalya Ghosh [1*]

[1] *Dept. of Physical Sciences, IISER- Kolkata, Mohanpur 741 246, Nadia, West Bengal, India*
[2] *Raja Ramanna Centre for Advanced Technology, Indore 452013, India*

Determination of the polarization parameters of the SLM using Mueller matrix measurements

A. Mueller matrix measurement strategy

The experimental system shown in Figure 1 of the manuscript was used to record the Mueller matrices of the SLM having different uniform grey level addressing. The Mueller matrix measurement strategy is based on sixteen intensity measurements performed by sequentially generating and analyzing four elliptical polarization states using a polarization state generator (PSG, comprising of a fixed linear polarizer P_1 with its axis oriented along the laboratory horizontal direction, followed by a rotatable quarter wave retarder QWP_1) and a polarization state analyzer (PSA, similar arrangement of fixed linear polarizer P_2 with its axis oriented along vertical direction and rotatable quarter wave retarder QWP_2, but positioned in a reverse order) unit respectively. The four required (and optimized) elliptical polarization states are generated by sequentially orienting the axis of the quarter wave retarder QWP_1 to four optimized angles, 35°, 70°, 105° and 140° with respect to the polarizer axis [1]. These four generated polarization states can be represented by a 4×4 matrix W, whose column vectors are the corresponding four generated Stokes vectors. After sample interaction (transmitted through the SLM), the resulting output polarization states of the transmitted light are analyzed in the PSA unit, by sequentially changing the orientation angle of QWP_2 to the same angles as PSG(35°, 70°, 105° and 140°). The PSA results can also be described by a 4×4 analyzer matrix A. The Stokes vectors of the light to be analyzed are projected onto the four basis states, given by the rows of A. The sixteen intensity measurements required for the construction of a full Mueller matrix are grouped into the measurement matrix M_i, which can be related to PSA/PSG matrices W and A, as well as the sample Mueller matrix M by [1,2]

$$M_i = AMW \qquad \text{(S1)}$$

Once the exact forms of the A and W matrices are known, the sample Mueller matrix can be determined as

$$M = A^{-1}MW^{-1} \qquad \text{(S2)}$$

The exact experimental forms of the W and A matrices were determined using the so-called Eigenvalue calibration method by performing measurements on calibrating samples having known forms of Mueller matrices (e.g., a set of quarter wave plates and polarizers) [1]. This approach enables one to correct for non-ideal behaviour of optical components, misalignments etc., and ensures high accuracy of Mueller matrix measurements. The details of the eigenvalue calibration method can be found in our previous publication [1] and elsewhere [3].

Mueller matrices were recorded from the SLM with different uniform grey level addressing (n). Illustrative example of Mueller matrices recorded from the SLM having three different uniform grey level addressing (n = 40, 80, 120) are shown in the figure below.

Addressed Grey Level (*n*)	Mueller matrix
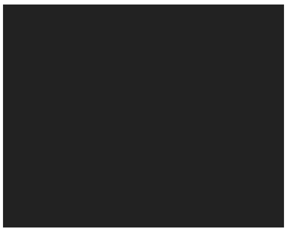 *n=40*	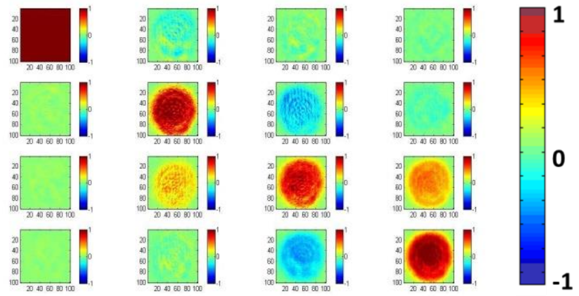
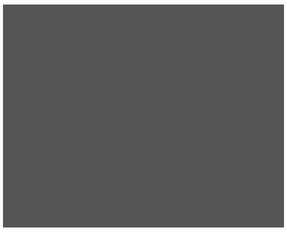 *n=80*	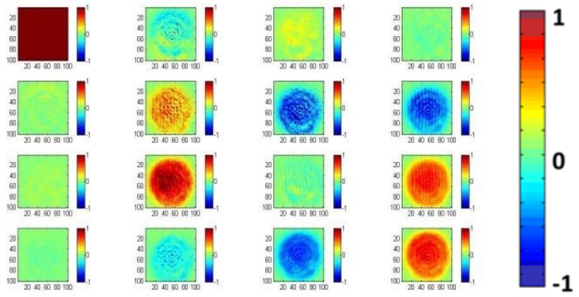
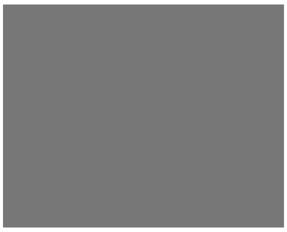 *n=120*	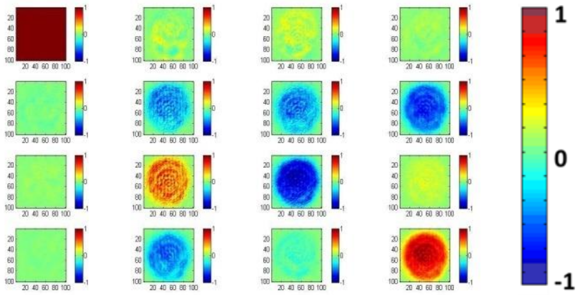

Figure: Full 4 × 4 Mueller matrix recorded from the SLM having three different uniform grey level addressing (n = 40, 80 and 120). The Mueller matrix elements are represented in normalized unit (normalized by the M_{11} element). The normalized values of the elements (between -1 to +1) are shown by the color bar.

The recorded Mueller matrices were analyzed subsequently to yield the polarization parameters of the SLM (effective linear retardance δ_{eff}(n) and optical rotation ψ_{eff}(n)) as a function of grey level (n), as described below.

B. Determination of the polarization parameters of the SLM

As shown and discussed in the manuscript (Figure 3a), the Mueller matrices of the SLM (with uniform grey level addressing) exhibited characteristic features of pure retarders exhibiting linear retardance and optical rotation effects and the contributions of other medium polarimetry effects (such as diattenuation and depolarization) were negligible. Even then, in order to eliminate any residual contributions of these polarimetry effects, the experimental

Mueller matrix M was decomposed into basis matrices of the three possible polarimetry effects [2, 4]

$$M \Leftarrow M_\Delta \bullet M_R \bullet M_D \qquad \text{(S3)}$$

with $\Leftarrow$ symbol used to signify the decomposition process. Here, the matrix M_Δ describes the depolarizing effects of the medium, M_R accounts for the effects of linear and circular retardance (or optical rotation), and M_D includes the effects of linear and circular diattenuation. The decomposition derived retardance matrix M_R was represented as a product of the basis matrices of an equivalent linear retarder (linear retardance δ_{eff} and orientation angle of the axis of the retarder θ_{eff}) and rotator (with optical rotation ψ_{eff}) (Jones → Mueller matrix conversion of Eq. 8 of the manuscript) [2,4]

$$M_R = M_{rot}(\psi_{eff}) M_{reta}(\delta_{eff}, \theta_{eff}) \qquad \text{(S4)}$$

where

$$M_{rot}(\psi_{eff}) = \begin{bmatrix} 1 & 0 & 0 & 0 \\ 0 & \cos(2\psi_{eff}) & -\sin(2\psi_{eff}) & 0 \\ 0 & \sin(2\psi_{eff}) & \cos(2\psi_{eff}) & 0 \\ 0 & 0 & 0 & 1 \end{bmatrix}$$

$$M_{reta}(\delta_{eff}, \theta_{eff}) = \begin{bmatrix} 1 & 0 & 0 & 0 \\ 0 & cos^2(2\theta_{eff}) + sin^2(2\theta_{eff})\cos(\delta_{eff}) & \sin(2\theta_{eff})\cos(2\theta_{eff})[1-\cos(\delta_{eff})] & -\sin(2\theta_{eff})\sin(\delta_{eff}) \\ 0 & \sin(2\theta_{eff})\cos(2\theta_{eff})[1-\cos(\delta_{eff})] & sin^2(2\theta_{eff}) + cos^2(2\theta)\cos(\delta_{eff}) & \cos(2\theta_{eff})\sin(\delta_{eff}) \\ 0 & \sin(2\theta)\sin(\delta_{eff}) & -\cos(2\theta_{eff})\sin(\delta_{eff}) & \cos(\delta_{eff}) \end{bmatrix}$$

Using the above forms of the linear retarder and optical rotator matrices, the values for effective linear retardance (δ_{eff}) and optical rotation (ψ_{eff}) can be determined from the matrix M_R as,

$$\delta_{eff} = \cos^{-1}(\sqrt{(M_R(2,2) + M_R(3,3))^2 + (M_R(3,2) - M_R(2,3))^2} - 1)$$

$$\psi_{eff} = \frac{1}{2}\tan^{-1}\left(\frac{M_R(3,2) - M_R(2,3)}{M_R(2,2) + M_R(3,3)}\right) \qquad \text{(S5)}$$

The results of determination of the polarization parameters of the SLM are summarized in Figure 3b of the manuscript.

References:

1. J. Soni, H. Purwar, H. Lakhotia, S. Chandel, C. Banerjee, U. Kumar, and N. Ghosh, "Quantitative fluorescence and elastic scattering tissue polarimetry using an Eigenvalue calibrated spectroscopic Mueller matrix system", Optics Express, 21, 15475 – 15489 (2013).
2. N. Ghosh and I.A. Vitkin, "Tissue polarimetry: concepts, challenges, applications and outlook'', J. Biomed. Opt.,**16**, 110801, (2011).
3. B. Laude-Boulesteix, A. De Martino, B. Drévillon and L. Schwartz, "Mueller polarimetric imaging system with liquid crystals," Appl. Optics, **43**, 2824 – 2832 (2004).
4. S. Y. Lu and R. A. Chipman, "Interpretation of Mueller matrices based on polar decomposition," J. Opt. Soc. Am. A, **13**, 1106 - 1113 (1996).
